# The surgical relevance of the anatomic position of the extraosseous mental nerve in a Kenyan population

**DOI:** 10.11604/pamj.2014.18.51.3831

**Published:** 2014-05-15

**Authors:** Poonamjeet Kaur Loyal, Fawzia Butt, Julius Alexander Ogeng'o

**Affiliations:** 1 Department of Human Anatomy, University of Nairobi, Nairobi, Kenya

**Keywords:** Mental nerve position, Kenya, anatomic position

## Abstract

**Introduction:**

Precise location of the mental nerve is important in implant surgery, administration of mental nerve block anaesthesia, and for osteotomy procedures. The position is known to show inter-population differences but data from sub-saharan region is scarce.

**Methods:**

The point of emergence of 64 nerves was studied and data analyzed with Microsoft Excel 2010 and differences in side means compared using the paired one tailed student's *t* test.

**Results:**

The location of left mental nerve was 2.85 (±0.38) cm, 1.42 (±0.33) cm, 1.77 (±0.46) cm while the right was 2.91(±0.47) cm, 1.38 (±0.3.1) cm, 1.71 (±0.46) cm from the mental symphysis, inferior border of mandible and cemento-enamel junction respectively. The differences in position between the right and left sides were not statistically significant (p< 0.05 CI: 95%). It emerged inferior to but in line with the 2^nd^ premolar in 57.8% of the cases, 2^nd^ premolar-1^st^ molar (25%) and inter-premolar junction (9.4%). Unique to this study, was the location of the MN at the canine (3.1%), and 1^st^ molar (4.7%) positions.

**Conclusion:**

The aberrant position of the mental nerve seen in 42%, is an important consideration for tooth implants and placement of mandibular reconstruction plates.

## Introduction

The mental nerve (MN) is the terminal branch of the inferior alveolar nerve. It exits the mental foramen (MF) near the 2^nd^ premolar [1] and divides into three branches to innervate the skin of chin, lower lip and gingivae. Most commonly, it lies below the midpoint of the distance between the lower border of the mandible and the alveolar margin. In the horizontal plane, it is approximately one quarter of the distance from the mental symphysis to the posterior border of the ramus of the mandible [2].

Anatomy of the extraosseous MN displays variations in position. The location of MF shifts from the near the lower border in a child to midway between the upper and lower borders of the mandible in an adult and then closer to the upper margin in the edentulous jaw of old age [3]. This position can be more accurately described in the vertical and horizontal planes for each age group [2, 4]. In adults, the MF lies below the midpoint of the distance between the lower border of the mandible and the alveolar margin and approximately one quarter of the distance from the MS to the posterior border of the ramus of the mandible [2]. This position is however, not conserved among the different populations and data for the Kenyan populace is lacking. The position can further be determined using teeth levels. Variant tooth levels of the MN include the 1^st^ premolar, interpremolar junction, junction of the 2^nd^ premolar and molar [5–7]. These parameters are important in locating the MN during surgical procedures involving the chin but such data is still not available for Kenyan patients. Precise location of the MN is important in placement of tooth implants, reconstructive surgery of the mandible and in avoiding iatrogenic injury during administration of anesthesia.

## Methods

This was a cadaveric dissection study of sample size 32 cadavers at the Department of Human Anatomy, University of Nairobi. Specimens from both sexes and of adult age group were studied. All the specimens were of indigenous Kenyan descent and of adult age group.

The MN was systematically exposed with three skin incisions and lateral reflection of the skin flap. A midline incision extended from the midpoint of the superior border of the lower lip to the midpoint of the inferior border of the MS. A lateral incision extended about 7cm from the angle of the lip on either side. An inferior incision was made along the lower border of the mandible from the MS till the lateral extent of the lateral incision. Using blunt dissection, the flap was detached from the bone and reflected laterally until the mental nerve was seen to exit from the mental foramen. The position was determined with respect to the MS, the cemento-enamel junction (CEJ) and the inferior border of the mandible using a Haco^®^ ruler in centimeters, inextensible string and a pair of dividers. The position with respect to the tooth level was also noted.

Data collected was analysed using Microsoft Excel 2007. The frequencies, means, percentages and standard deviations of the morphometric data were calculated and frequencies and percentages were determined. The statistical significance of side differences was determined using the paired one tailed student's *t* test. A p-value of <0.05 was considered to be statistically significant.

## Results

The MN was on average, 2.89 (±0.42) cm, 1.40 (±0.38) cm and 1.75 (±0.38) cm from the MS, inferior border of mandible and CEJ respectively. The location of left mental nerve was 2.85 cm, 1.42cm, 1.77cm while the right mental nerve was 2.91cm, 1.38cm, 1.71cm from the MS, inferior border of mandible and CEJ respectively. The results, although, were not statistically significant, the left MN was observed to be closer to MS while the right was closer to the inferior border of the body of the mandible and the CEJ. In 57.2%, the MN was coincident with the 2^nd^ premolar. The second most common tooth position was observed in 25.9%, in line with the junction between 2^nd^ premolar and 1st molar. Less frequently, it was also seen to emerge at the level of inter- premolar junction in 9.4%, the 1^st^ molar in 4.7% and canine in 1.8% of the cadavers.

## Discussion

The MN was on average, 2.89 (±0.42), 1.40 (±0.38) and 1.75 (±0.38) cm from the MS, inferior border of mandible and CEJ respectively. The average distance of the MN from the MS and the inferior border of the mandible is similar to that reported by previous studies (Table 1). The distance from the tooth is, however at variance compared to that reported for the Thai and Sri Lankan population [8, 9]. This may be due to the differences in the superior landmarks used i.e. whereas the current study employed the cemento-enamel junction, the Thai study used the tip of the buccal cusp of the 2^nd^ premolar as the superior boundary.

**Table 1:** Position of the MN in different populations

Study	Population	n	MS (cm)	CEJ/buccal cusp of 2^nd^ premolar (cm)	Inferior border of mandible (cm)
**Current study, 2012**	Kenyan	64	2.89	1.75	1.40
***Apinhasmit*** ***et al*****., 2006**	Thai	138	2.883	2.427	1.488
***Prabodha and Nanayakkara***, **2006**	Sri Lanka	48	2.652	-	1.225

The location of left mental nerve was 2.85 cm, 1.42cm, 1.77cm while the right mental nerve was 2.91cm, 1.38cm, 1.71cm from the MS, inferior border of mandible and CEJ respectively. The discrepancies between results of the current study and those reported in previous studies [10–12] (Table 2), could be attributed to differences in sample sizes, intraobserver errors while measuring and differences in the landmarks used. The measurements for previous studies were done using the MF but the current study used boundaries of the MN as landmarks. Furthermore, the superior boundaries also differed with previous studies using the alveolar margin and the current study using the CEJ.

**Table 2:** Comparison of the position of the MN with previous studies

			Landmark		
	Study population	Side	MS (cm)	Superior boundary (cm)	Inferior border (cm)
**Current Study (n=64)**	Kenyan	Left	2.85 ±0.38	1.77 ±0.46	1.42 ±0.33
		Right	2.91 ±0.47	1.71±0.46	1.38 ±0.31
Igbigbi and *Lebona* *et al*., (2005) (n=149)	Malawian	Left	2.631±0.431	1.296±0.343	1.349±0.355
		Right	2.649±0.463	1.377±0.355	1.324±0.283
*Agarwal and Gupta*, 2011 (n=100)	Indian	Left	2.505±0.507	1.382±0.306	1.211±0.311
		Right	2.555±0.507	1.405±0.305	1.216±0.304
*Yesilyurt* *et al*., 2008 (n=140)	Turkish	Left	1.937±1.133	1.064	0.946±0.5877
		Right	1.918±1.119	1.050	0.944±0.5837

The MN is difficult to localize as there are no absolute landmarks. These distances, therefore, are relevant in approximating the position of the MN during the placement of mandibular reconstruction plates, tooth implants, and harvesting bone from the MS which will be used to fill for craniofacial defects. The location of the MN is also important to an anesthetist when performing a MN bock so as to achieve effective anesthesia and at the same time avoid iatrogenic injury to the nerve. These distances are especially important when teeth are missing or mal-positioned and hence cannot be used to approximate the position of the MN

The most common tooth position for the MN was in line with the 2^nd^ premolar (57.2%). This is consistent with results of previous studies [7, 13, 14] that have reported the prevalence of 45-68.6% for locating the MN at 2^nd^ premolar (Table 3). The second most common position was between the junction of the 2^nd^ premolar and the 1^st^ molar, which was noted in 25.9%. This was similar to other studies from East Africa done on dry mandibles [7, 13]. It was, however, at variance with the studies from Nigeria [15] and India [14] that have reported the interpremolar junction as the 2^nd^ most common position for locating the MN. Unique to this study, was the aberrant position of the MN at the level of the canine. These discrepancies in the prevalence might be as a result of differences in the sample sizes used by these studies.

**Table 3:** Prevalence of the different tooth positions amongst populations

Study (n)	Population	Tooth Position					
		2^nd^ Premolar	Junction:2^nd^ premolar and 1^st^ molar	Interpremolar	1^st^ Molar	Canine	Mesial to 1^st^ premolar
**Current study, 2012 (64)**	Kenyan	57.8	25.9	9.4	4.7	3.1	-
***Singh and Srivastav*****, 2010 (100)**	Indian	68.6	11.5	17.8	-	-	-
***Fabian*****, 2007 (100)**	Tanzanian	45	35	12	8	-	-
***Mwaniki and Hassanali*****, 1992 (100)**	Kenyan	56.1	31.1	12.8	-	-	-
***Kekere-Ekun*****, 1989 (604)**	Nigerian	55.63	12.3	26.99	3.3	-	0.17

The tooth position is a quick landmark for locating the MN. Whereas, surgeons should be aware that the most common location for the MN is at the 2nd premolar, this however, should not be the sole guidance, since in 41.8% the MN may have an aberrant tooth position. Location of the MN at an aberrant tooth position could also help in explaining cases of failed anesthesia, when it is targeted at the 2^nd^ premolar region. In such cases the junction of the 2^nd^ premolar and 1^st^ molar should be considered as a potential alternative site for the MN.

## Conclusion

The position of the mental nerve which was on average, 2.89 (±0.42) cm, 1.40 (±0.38) cm and 1.75 (±0.38) cm from the mental symphysis, inferior border of mandible and cemento-enamel junction respectively, is an important consideration for tooth implants and placement of mandibular reconstruction plates. In 42%, it occurred at an aberrant tooth position which may explain the occurrence of failed anaesthesia.
